# Supplementary material for: Cysteine Mutants of the Major Facilitator Superfamily-Type Transporter CcoA Provide Insight into Copper Import
Source: mBio. 2021 Jul 20;12(4):e01567-21. doi: 10.1128/mBio.01567-21 (PMC8406296; doi:10.1128/mBio.01567-21)
Supplement: TABLE S1 [file mbio.01567-21-st001.docx]

**Table S1. Strains and Plasmids**

| **Plasmids / strains** | **Description** | **Antibiotic**  **Resistance** | **Reference** |
| --- | --- | --- | --- |
| ***Strains***  *E. coli*  HB101 | F^-^ Δ(*gpt-proA*)62 *leuB6 supE44 ara*-14 *galK2 lacY* 1 Δ (*mcrC-mrr*) *rpsL20* (Str^R^) *xyl-5 mtl-1recA*13 | Str^R^ | Promega |
| LMG194 | F-∆l*acX74 galE thi rpsL ∆phoA* (Pvu II) ∆*ara714 leu::Tn10* | Tet^R^ | Invitrogen |
| *R. capsulatus*  SE8 | ∆*ccoA* | Spe^R^ | ([1](#_ENREF_1)) |
| ***Plasmids***  pBluescript II | Cloning vector (pBS) | Amp^R^ | Stratagene |
| pRK2013 | Conjugation Helper | Kan^R^ | ([2](#_ENREF_2)) |
| pRK415 | Broad-host-range vector | Tet^R^ | ([2](#_ENREF_2)) |
| pBAD/Myc-His(A) | Arabinose inducible cloning vector | Amp^R^ | Invitrogen |
| pBK68 | 1.2 kb of *ccoA* in pBAD/Myc-His (A) | Amp^R^ | ([3](#_ENREF_3)) |
| pBK69 | 1.2 kb of *ccoA* in pBAD/pRK415 | Amp^R^ Tet^R^ | ([3](#_ENREF_3)) |
| pSP7 | *ccoA::M237A+M265A* in pBAD/Myc-His | Amp^R^ | This work |
| pBK98 | *ccoA::M30A* in pBAD/Myc-His | Amp^R^ | This work |
| pBK99 | *ccoA::M32A* in pBAD/Myc-His | Amp^R^ | This work |
| pBK100 | *ccoA::M69A* in pBAD/Myc-His | Amp^R^ | This work |
| pBK101 | *ccoA::M73A* in pBAD/Myc-His | Amp^R^ | This work |
| pSP6 | *ccoA::M227A* in pBAD/Myc-His | Amp^R^ | This work |
| pSP4 | *ccoA::H249A* in pBAD/Myc-His | Amp^R^ | This work |
| pSP5 | *ccoA::H274A* in pBAD/Myc-His | Amp^R^ | This work |
| pBK108 | *ccoA::C49A* in pBAD/Myc-His | Amp^R^ | This work |
| pBK109 | *ccoA::C109A* in pBAD/Myc-His | Amp^R^ | This work |
| pSP9 | *ccoA::C225A* in pBAD/Myc-His | Amp^R^ | This work |
| pSP8 | *ccoA::C247A* in pBAD/Myc-His | Amp^R^ | This work |
| pBK117 | *ccoA::C367A* in pBAD/Myc-His | Amp^R^ | This work |
| pBK119 | *ccoA::C49A+C109* in pBAD/Myc-His | Amp^R^ | This work |
| pBK122 | *ccoA::C49A+C247A* in pBAD/Myc-His | Amp^R^ | This work |
| pBK126 | *ccoA::C109A+C247A* in pBAD/Myc-His | Amp^R^ | This work |
| pBK123 | *ccoA::C247A+C367A* in pBAD/Myc-His | Amp^R^ | This work |
| pBK129 | *ccoA::C109A+C367A* in pBAD/Myc-His | Amp^R^ | This work |
| pBK95 | *ccoA::M237A+M256A* in pBAD/pRK415 | Amp^R^ Tet^R^ | This work |
| pBK102 | *ccoA::M30A* in pBAD/pRK415 | Amp^R^ Tet^R^ | This work |
| pBK103 | *ccoA::M32A* in pBAD/pRK415 | Amp^R^ Tet^R^ | This work |
| pBK104 | *ccoA::M69A* in pBAD/pRK415 | Amp^R^ Tet^R^ | This work |
| pBK105 | *ccoA::M73A* in pBAD/pRK415 | Amp^R^ Tet^R^ | This work |
| pBK92 | *ccoA::M227A* in pBAD/pRK415 | Amp^R^ Tet^R^ | This work |
| pBK90 | *ccoA::H249A* in pBAD/pRK415 | Amp^R^ Tet^R^ | This work |
| pBK91 | *ccoA::H274A* in pBAD/pRK415 | Amp^R^ Tet^R^ | This work |
| pBK111 | *ccoA::C49A* in pBAD/pRK415 | Amp^R^ Tet^R^ | This work |
| pBK112 | *ccoA::C109A* in pBAD/pRK415 | Amp^R^ Tet^R^ | This work |
| pBK94 | *ccoA::C225A* in pBAD/pRK415 | Amp^R^ Tet^R^ | This work |
| pBK93 | *ccoA::C247A* in pBAD/pRK415 | Amp^R^ Tet^R^ | This work |
| pBK120 | *ccoA::C367A* in pBAD/pRK415 | Amp^R^ Tet^R^ | This work |
| pBK121 | *ccoA::C49A+C109A* in pBAD/pRK415 | Amp^R^ Tet^R^ | This work |
| pBK124 | *ccoA::C49A+C247A* in pBAD/pRK415 | Amp^R^ Tet^R^ | This work |
| pBK127 | *ccoA::C109A+C247A* in pBAD/pRK415 | Amp^R^ Tet^R^ | This work |
| pBK125 | *ccoA::C247A+C367A* in pBAD/pRK415 | Amp^R^ Tet^R^ | This work |
| pBK130 | *ccoA::C109A+C367A* in pBAD/pRK415 | Amp^R^ Tet^R^ | This work |

**References for Table S1 and Table 2**

1. Ekici S, Yang H, Koch HG, Daldal F. 2012. Novel transporter required for biogenesis of *cbb*_3_-type cytochrome *c* oxidase in *Rhodobacter capsulatus*. *MBio* **3**.

2. Ditta G, Schmidhauser T, Yakobson E, Lu P, Liang XW, Finlay DR, Guiney D, Helinski DR. 1985. Plasmids related to the broad host range vector, pRK290, useful for gene cloning and for monitoring gene expression. *Plasmid* **13**:149-53.

3. Khalfaoui-Hassani B, Verissimo AF, Koch HG, Daldal F. 2016. Uncovering the Transmembrane Metal Binding Site of the Novel Bacterial Major Facilitator Superfamily-Type Copper Importer CcoA. *MBio* **7**:e01981-15.
