# Supplementary material for: Cysteine Mutants of the Major Facilitator Superfamily-Type Transporter CcoA Provide Insight into Copper Import
Source: mBio. 2021 Jul 20;12(4):e01567-21. doi: 10.1128/mBio.01567-21 (PMC8406296; doi:10.1128/mBio.01567-21)
Supplement: FIG S1 [file mbio.01567-21-sf001.pdf]

Figure S1. CcoA of Rhodobacteriales (see legend below)

```
Rubrimonas          ---mahastpaald--aaqpahddakarrnvavlvaaqallgaqmpihfilgglagqlla     55
Roseibacterium      -------mtdasl---tpediagdsrarrnvavlvaaqailgsqmpmiftvgalaglmia     50
Paracoccus          -----------------------mdrarrnvavlvaaqailgaqmsinfiigglagqila     37
                                                                  30 32
capsulatus          ---------------MTQTAASAPPLPLRNLIVLVAAQAFLGAQMSMIFTVGGLAGQSLA     45
Rhodovulum          ------------mtd-tlpspavsprarrnvtvlilaqallgaqmpmiftvgglagqtla     47
Thalassobius        -------mtdaintpapdtgptsdaaakrnvvilvlaqailgaqmpmlftigglagqsla     53
sphaeroides         ------------MTDASALPA-SDARARRNVIVLVAAQAILGSQLSMVFIVAGLSGQTLA     47
Gemmobacter         ------------mtvlaappapddrlarrnvfvlvaaqailgaqmpmiftiaglagqsla     48
Roseivivax          mrhlaadrrpayrcsmt--aaatpvrarrnvavlvaaqailgsqmpmiftigglagqsla     58
Actibacterium       ------------mtqat--apydpararrnvtvlvlaqallgsqmamiftvgglagqsla     46
Roseovarius         ------------mtdav--lphsdtrakrnvavlvlaqavlgsqmpmifvvgglagqsla     46
                                                    **: :*: ***.**:*: : * :..*:*  :*

Rubrimonas          tnkalatlpiaatvamamvaaplvsmlmgrvgrrpgfllgalcgalggavsaaallvgsf     115
Roseibacterium      pspalatipislivfgsmttalwlsplmqrfgrragffigavagaigaaisayglwsnsf     110
Paracoccus          pnpciatlplsmivlgsalaaqplsgfmqrhgrragfllavgagasgaalsaaglwigsf     97
                       49               69  73
capsulatus          TNPCLATLPLSLIVLGSVLTAQPMSSFMAVYGRRAGFILATAAGGIGAAISAHALAIGSF     105
Rhodovulum          pnaclatlpislivlgsmltagplsalmqrwgrragfvlgvaggaagagasalglylgsf     107
Thalassobius        snicfatlpitmivlssmlsatpvaavmqrfgrrvgfmmgtafgaagavvgayalsiasf     113
sphaeroides         SNPCWATLPITMAVTGSMLSATPLSAIMQRFGRRVGFLVGALAGALGAAVCAVALMQGSF     107
Gemmobacter         snpcwatlpisatvlgsmlsatplsalmqrhgrragfvagavggmvgaaigafalyignf     108
Roseivivax          pnpcfatlpislivlgsmlaaqplsslmqrvgrragflvgvtggamggaigaaglytqsf     118
Actibacterium       pnacfatlpismivlgsmltatplssfmqkagrragffaggaggaigaavcayalmqnsf     106
Roseovarius         snicfatlpislivlgsmlsatplswamqrwgrragfvigtaggaigatigayglylgsf     106
                     . . **:*::  *  :  :*  ::  *   *** **. .   *  *.   * .*   .*

Rubrimonas          pmlvagaaltgvymgaqgfyrfaaadaaspafrpraiswvmaggllaaligpelvkltrd     175
Roseibacterium      alflagsyvtgiymsaqgfyrfaaadtasenfrpkaisyvmagglisaligpqmvklttd     170
Paracoccus          wlfmagslltgiymaaqgfyrfaatdtappdfaaraiswvmaggllsaiigpavvrltnd     157
                       109
capsulatus          PLFCLGSLLAGIYMSAQGFYRFAATDGIAPEHQSKAISWVLAGGLAAAVLGPQLVKLTAQ     165
Rhodovulum          plfllgalltgiymsaqgfyrfaaadtaegtfqpkaisyvlagglaaailgpqlvkvtae     167
Thalassobius        plflvgsfltgtymsahgfyrfaaadtasdafrpkaisyvmagglaaaiigpqlvkltsd     173
sphaeroides         ALFLAGSLLTGTYMSAQGFYRFAAADTASETFRPKAISWVMAGGLLSAVIGPQVVKLTAE     167
Gemmobacter         plfvlgalftgmymsaqgfyrfaaadtasedfrpkaiswvmaggllsaiigpqlvkitad     168
Roseivivax          wlfllgslftgiymaaqnfyrfaatdtaseefrpkaisyvlagglaaaligpqivkltae     178
Actibacterium       tlflvgsfftgiymsaqgfyrfaaadtasedfrpkaisyvlagglasailgpqlvkltst     166
```

```
Roseovarius          pvyllgslftgmymsaqgfyrfaaadtasdefrpraisyvlagglisavigpqlvkmtae    166
                      :   *: .:* **.*:.******:*     .  :***:*:**** :*::** :*::*

Rubrimonas           alaptpfagaylsmvalnllgacafifldaprppkpaa---dapqarplsvivrqprvav    232
Roseibacterium       at-vipflgsyvfvvlinlvgmalflfldlpkptaahraatragagrsyaellrdpriav    229
Paracoccus           lt-avpfmasyaavivlnltgpflfafldipkppapgv--vgetggrqlremlrvpqigv    214
capsulatus           AL-VVPFQATYLAIIAINLAGPLIFAFLRIPAPGRRVK---GQAGGRTRGELLRDPVILV    221
Rhodovulum           tm-vipflgtyltaiainlggglfafldiprppprdp---ahhagrtraellrdpviav    223
Thalassobius         tf-vvpflgayvaiiainllgsllflfldiptppkpsa---dapkgrtrwqlittpviav    229
sphaeroides          AM-VVPFLGTYLAAILLNLCGVALFAFLDIPRPTPPAA---GSPRGRSRLELLREPRIAV    223
Gemmobacter          af-vfpflgtylavialnavgmflfaglriptpqppsa---dtprgrtrmellrtpviav    224
Roseivivax           af-vvpflgtyaaviavnlvgasvflfldiprppkpda---daprgrsvpqllaspriav    234
Actibacterium        sm-vvpflgtyaaviainvigsflfvfldipkpaapaa---dapqgrsrlellrtptiav    222
Roseovarius          am-vipflgtylavigvnlvgaflflfldipkppvpgd---daprgrsrmellttpriav    222
                          ** .:*   : :*  *   *  *  * *            .*    ::  * : *

                        225 227     233  235         247 249         261   265        274
Rubrimonas           amicamvtyalmnlvmtstplavvacgfdpddaasvvkwhvlamfapsfftgdliarfga    292
Roseibacterium       aiicgmvsyalmnlmmtstplavvgcgfttgsaadvvsahviamfapsfvtghliarfga    289
Paracoccus           amicgmvsyalmnlvmtstplavvgcgfapenaadivsahvlamfvpsfftghlivrfga    274
capsulatus           AMICGMVSYALMNLVMTSTPLAVVGCGHTTTNAADIVSAHVLAMYLPSFFTGHLIARFGR    281
Rhodovulum           aiicgtvsyalmnlvmtstplavvgcgfdtgtaadivtahvlamyvpsfftghliarfga    283
Thalassobius         avicgmvsyalmnlvmtstplavvgcgfeqdnaadivtahvlamyipsfftghliarfgt    289
sphaeroides          AVICATVAYALMNLVMTSSPLAVVGCGFATSDAANVVTAHVLAMYGPSFFTGHVIARFGA    283
Gemmobacter          avicatvsyalmnlvmtssplavvgcgfetgdaanvvtahvlamyapsfitghiiarfga    284
Roseivivax           avicamvsyalmnlmmtstplavvgcgfttgsaadvvsahviamfapsfftghliarfga    294
Actibacterium        aiicalvsyalmnlvmtstplavvgcgfetddaanvvsahvlamfvpsfftghliakfgv    282
Roseovarius          avicatvsyalmnlvmtstplavvgcgfetsnaadvvtghvlamfapsfftghliarfgt    282
                     *:**. *:******:***:*****.**.    **.:*. **:**: ***.**.:*.:**

Rubrimonas           priialglamlaacggvaltgvdltqfygalillglgwnfgfigatamlaaahrpeersr    352
Roseibacterium       mrivalglfllalagavalsgvelyqffgalvllgvgwnfgfigatamltqahevhergr    349
Paracoccus           erivglgliiltlagavalsgvelghffgalillgvgwnfgyigatamltrahrpeergr    334
capsulatus           ETIVGIGLFILAVAGAVALTGVDLEQFFLALMLLGLGWNFGFIGSTAMLAAAHAPEERGT    341
Rhodovulum           rpivalglailagagtvaltgveieqffaalvllgigwnfgfigatamlssahgpeerga    343
Thalassobius         qkiiaaglvilagaglvalqgvelenffialvllgigwnfgfigatsllaashtveergr    349
sphaeroides          EKVMALGLAILAGSGAVALSGVELGHFFGALVLLGVGWNFGFIGATALLASAHAPEERGR    343
Gemmobacter          dkvvamglailagagavaltgvelekffialillgvgwnfgfigattmlassyaphergr    344
Roseivivax           privgaglvclaaasatglagvdlenffgalvllglgwnfgfigatsmlaaahtleergr    354
```

```
Actibacterium       ekiiatglvilagagavalsgvdiehffialvllgvgwnfgfigatamlagahephergk     342
Roseovarius         ekimglglfilacagavalqgvelenfflalillglgwnfgfigattmlaashephergr     342
                      ::. **  *: .. ..* **:: :*: **:***:*****:**:*::*: ::  .**.

Rubrimonas          vqglndflvfgmvtvaslssgalmn------dlgweavnlamipflalagaallwlaiar     406
Roseibacterium      vqglndflvfgcvtiaslasgflms-sgtdtqagwtavniamipflalaggaliwlalrp     408
Paracoccus          iqgvndffvfggvflaslssgglmnclggsvqagwnavnlamlpflclagaaliwlmmrp     394
                                                 367
capsulatus          VQGMNDFVVFGGVFLASLSSGGLMTCASADAVAGWQAVNLAMLPFLTLAGAALIWLVLRP     401
Rhodovulum          vqgmndilvfggvtmaslasgglmncsggvpqegwvavnlamvpflalaggaliwlvlrp     403
Thalassobius        mqglndllvfggvtlaslasgglmncsggeaavgwaavniamapflmlaggaliwlmfqp     409
sphaeroides         IQGLNDLIVFGGVAMASLASGGLMNCSGGSVEAGWQAVNLAMLPFLVAAGGALIWLALRP     403
Gemmobacter         mqglndlivfggvtlaslssgglmncsggsvqsgweavniamvpflvlsggaliwlmarp     404
Roseivivax          aqgmndvivfggvtmaslasgglmncsggtaqegwaavnlamapliclaaaallwgvmrp     414
Actibacterium       vqgmndlivfggvtlaslasgglmncsggsveqgwasvnlamipflmlaggaliwltlrp     402
Roseovarius         mqglndlivfggvtmaslssgglmncsggtpeagwasvnlamipcltlaggaliwlvlrp     402
                     **:**..*** * :***:** **.        ** :**:** * :  :..**:*

Rubrimonas          ppaea-   411
Roseibacterium      rdmlv-   413
Paracoccus          ees---   397
capsulatus          KDTR--   405
Rhodovulum          gdkd--   407
Thalassobius        keema-   414
sphaeroides         KEA---   406
Gemmobacter         kaa---   407
Roseivivax          rtapqg   420
Actibacterium       keav--   406
Roseovarius         kea---   405
```

Figure S1. Alignments of CcoA amino acid sequences among Rhodobacterales species. The CcoA sequences are from *Rhodobacter capsulatus* (**capsulatus**), *Rhodobacter sphaeroides* (**sphaeroides**), *Paracoccus alcaliphilus* (Paracoccus), *Roseibacterium elongatum* (Roseibacterium), *Actibacterium atlanticum* (Actibacterium), *Gemmobacter megaterium* (Gemmobacter), *Rhodovulum sulfidophilum* (Rhodovulum), *Roseivivax atlanticus* (Roseivivax), Roseovarius indicus (Roseovarius), *Thalassobius mediterraneus* (Thalassobius), *Rubrimonas cliftonensis* (Rubrimonas). The Met, His and Cys residues of interest are highlighted in yellow, green, and purple, respectively.
