# Supplementary material for: Cysteine Mutants of the Major Facilitator Superfamily-Type Transporter CcoA Provide Insight into Copper Import
Source: mBio. 2021 Jul 20;12(4):e01567-21. doi: 10.1128/mBio.01567-21 (PMC8406296; doi:10.1128/mBio.01567-21)
Supplement: FIG S2 [file mbio.01567-21-sf002.pdf]

Figure S2. CcoA of Proteobacteria (see legend below)

```
Thalassospira          ------------msspevrrnilflslcvalsasamsllftvaavigyslapdkslstlp   48
Synechococcus          ----------msavassvksqvtllalcqalamttntvlittaalvgyalatdkslatlp   50
Halomonas              -----------------mpfnvmllslcqallvsgnilliavspligarlapdpawstap   43
Vibrio                 -----------------mnrnvwllslcqallmtgnillisviglvgkaiapssslitlp   43
Pseudoalteromonas      -----------------mnknvfllaccqglmttgnillvtisalvgqklsasdtlitlp   43
                                                          30 32                 49
capsulatus             MT------QTAASAPPLPLRNLIVLVAAQAFLGAQMSMIFTVGGLAGQSLATNPCLATLP   54
Thioalkalivibrio       ---------msgqttpreranvlilalgqalfliaavtvmtlsgvvgerlasdpslatlp   51
Methylobacterium       msnpaaplpgtslpaatgtgnvlrlavaqalagansvvvyatgavigsrlapdpalatlp   60
Bordetella             -----mpdltgilpdrvqrgniwrlsiaqalagansvvvyatgaivgdmlapspmlatlp   55
Ochrobactrum           -------M----TDATAARRNIVILTIAQALGASSPPIVISLGGLVGQKLSSDPALVTLP   49
Pseudomonas            -----mpp----paatdarrnvgllmaaqslggaappiiislggivgqmlasnpslatlp   51
                                               :: *   .:     : :  : *  :: .   * *


Thalassospira          vaftmiammvttapsalmmnrfgrrvgfwigsvigmlga-vsgmgtvyfgsfwllcvaca   107
Synechococcus          lairqvatmsatipasvlmqrvgrrngfllgslvgiaga-glsiysltitsywlfalsma   109
Halomonas              vatqwlglmcatipaslimarlgrkrgfllgnllglagv-vvaaqalvtetftlfmlgtw   102
Vibrio                 valqflglmsatipaslimgklgrrkgfsignligifga-svatyalsqemfylfcvgtf   102
Pseudoalteromonas      iatqmlglllatvpaslvmaktgrklgfsmgnligtsga-ilgylalinsqfylfclatf   102
                                     69  73                                 109
capsulatus             LSLIVLGSVLTAQPMSSFMAVYGRRAGFILATAAGGIGA-AISAHALAIGSFPLFCLGSL   113
Thioalkalivibrio       vaammigtlvitlpaslfmkrfgrrtgfllgagvggfggaaiaavgvfaeafwifvlgnl   111
Methylobacterium       isifvvgmaactlpagriaraygrrtaflagtgsgvlvg-llaalavvlssfwlfclatf   119
Bordetella             isifvvgmaacilpagaiarrhgrraaflagtlagvltg-llamaavimggfwlfclatf   114
Ochrobactrum           VSLFNLGLALGTLPAAFFMRQFGRRNAYMLGALVGAAAG-VIAAAGIFAASFLIFCLGTL   108
Pseudomonas            vslynlglalstipaallmrrlgrraayalgallgsvsg-liaalgvlwgsfetfcvgta   110
                       ::   :.      *  .  .    **: .:  .   *       .   :    :  : :.


Thalassospira          cigsanaiamqyrfaaaeaappefrsraisltmlgglaaafvgpnlasfarnwfetvpfl   167
Synechococcus          llglsnslvgyyrfaaadvadealrsqaiswviaggiiaavlgpwlangskdwfdseiyi   169
Halomonas              ligigigfgqlyrfaaveaaplalrdraiglvmgggvlaaffgpwlarisre-aavtpfl   161
Vibrio                 llgigigfgtlyrfaaievcdesarhraisismaggvlaailgpnlaiysqqwsadglyv   162
Pseudoalteromonas      ligigigfatlyrfaaievsdkpa--raismimasgviaavlgpnlavwvnhylpeinfa   160
capsulatus             LAGIYMSAQGFYRFAATDGIAPEHQSKAISWVLAGGLAAAVLGPQLVKLTAQAL-VVPFQ   172
Thioalkalivibrio       llgayqafamyyrfaaadvasdafrpraislvmaggvaaaflgpwnahhaqallpqtpea   171
Methylobacterium       fggayaavvlsfrfaaadcvaperrpralsavmaggvfagiigpqlvshtmslwpshafa   179
Bordetella             fggcyaavvlsfrfaaadgvaperraralslvmaggvaagvvgpqlvtwtmdwwpphmfa   174
Ochrobactrum           TAGFYASYVQSYRFAATDAATGDMKARAISWVMVGGLVAAIVGPQLVIWTRDTIPDAMFA   168
```

```
Pseudomonas            magfygacvqsyrfaasdavpppqratvisrimiggliaavigpqvviwtrdawpmapfa   170
                         *    .    :**** :          .:.   :  .*: *...**   .

Thalassospira          gtfvaliglqfllviaigqlrlpdmrgt--khdeparplkmvigqpaviiamiagalgya   225
Synechococcus          gafvavlglqilsalllcflqiphssrr--klqtssrslfeivrqpkfgvatfgstisyg   227
Halomonas              gsfiglgvlylvallvllatrlppaeqt--hgdgqprplgeilrqpvfvvavlsaligyg   219
Vibrio                 gafmaliglnilalcllqtiqfpptheh--hqhaqpeplleivkapnfmlavfaamvaya   220
Pseudoalteromonas      nsflaltcvyllaltllqlvafneisgq--qslfqqrpikliitqpqfmiaafigmisys   218
                                                                         225 227
capsulatus             ATYLAIIAINLAGPLIFAFLRIPAPGRRV-KGQAGGRTRGELLRDPVILVAMICGMVSYA   231
Thioalkalivibrio       gpylllgalallamlllawlrvptppa---aeraisrplreimprgrfvvalttaalgya   228
Methylobacterium       atflaqaavavlsavvlmgvrlpgptr---aeisggrplgtiarqprfvtavlcgvvsyl   236
Bordetella             atflvqaavaaasalillgvrlpmpga---aemasgrplaeiarqprfvaavvcgavsym   231
Ochrobactrum           GSFLSQAVLGLLALPVLFMLRAPKVRKDPNAIHDTGRPLGEILRSPRFILSVAAGVCSYA   228
Pseudomonas            gsflgqaglallalplllmlrmpppq--asavvgaarplaviarspgfvvavtagivsyg   228
                         ::    :       :                   .     :     .  :    .   .*


Thalassospira          vmsfvmtatplaildcnyefgdaafiiqwhvvgmyapgfftgnlirrfgaltiiqvgavl   285
Synechococcus          vmafvmtatplamsaenhsfsqsaaviqwhvlgmfgpalitgwlikrlgvlsiiltgaal   287
Halomonas              vmnlamtatplamagaghhfdhvattiqwhvlamflpsfftghltarfgaprmivagcvl   279
Vibrio                 vmnilmtatplamigcgfdfekaagviewhvlgmfvpafftgrlierfgarnmiisggil   280
Pseudoalteromonas      vmnllmtatplamhrhgfdladsalviewhvlgmflpsfftgklvekytpvavimigcll   278
                        233   237      247 249          261   265        274
capsulatus             LMNLVMTSTPLAVVGCGHTTTNAADIVSAHVLAMYLPSFFTGHLIARFGRETIVGIGLFI   291
Thioalkalivibrio       vmillmtatplamrqagfdmgqvalvmqwhvlgmfapsfvtghlitrfgltrvlalgnal   288
Methylobacterium       lmnflmtaaplamhlcgftqadanlglqwhviamyapsfvtgrliarfgapsvvaaglal   296
Bordetella             lmnflmtaaplamhlcghsqqsanlglqwhviamyapsfftgslivrfgalrmataglvl   291
Ochrobactrum           LMTFVMTAAPIAMVGHGHSVDHAALGIQWHVLAMFAPSFFTGKLITRFGKEKITALGLVL   288
Pseudomonas            lmafimtaapmamvgcghtvgeaalgiqwhvlamfapsfftghliarfgktaitacglvl   288
                       :* : **::*:*:   ..         :. **:.*: *.:.** *  :     :   *  :


Thalassospira          nliclafglagedlignfwpslvllgvgwnfmfvgattfltenyrpaeqarvqainefvv   345
Synechococcus          llgcmglnlagtsf-whfaialllglgwnfmyvgsttlltetytsdekaqaqavhdfiv   346
Halomonas              laasglaaqveagv-agfhfalillglgwnftflpatglltetyrpvekartqaaneflv   338
Vibrio                 fvlciainihgqsi-whfslalillgvgwnfmfiaatglfsqsysprnkskaqafneffv   339
Pseudoalteromonas      mlgcaiinllgvsh-whflialfllgvgwnfmfisatslvsetyrpherakaqasnefvv   337
capsulatus             LAVAGAVALTGVDL-EQFFLALMLLGLGWNFGFIGSTAMLAAAHAPEERGTVQGMNDFVV   350
Thioalkalivibrio       vlfsvaiaahgetl-ahfwlalfllgvgwnflfiggstlltttthtagekgkvqgvndltv   347
Methylobacterium       tataaivglsgldl-ahfwaflvllglgwnfgfvgasamvlechrpeertrvqslndfvv   355
Bordetella             tglsaaiglagvdv-ahfwwslillglgwnfgflgasalvlechrpeektrvqslndfiv   350
Ochrobactrum           IAFSAIIALGGFDV-GHFWGALIFLGIGWNFGFIGATAMVTDCHTPAERGKAQGANDFIM   347
```

```
Pseudomonas              igtsgllalaglel-lhfwgslillgvgwnfgfigatalvtdcytaperakvqalndflv   347
                            .             *   *.:**:**** :: .: :.   :   ::  .*. ::: :


Thalassospira            fgtvavaslsagsiya------gagwatllysaalpvglvmlvlagyalryrrqha----   395
Synechococcus            fgfvaiatyssgqify------sfdwvmln-qiswplvlavlltvlwlqnqlissnqr--   397
Halomonas                fstvavtallagplvn------qlgwalln-allmplslvpiallgwqrlarrqdphis   391
Vibrio                   fscvtvtallsgwles------tvgwemmn-iyvlpfvlvvlalfgvntfrsrqsia---   389
Pseudoalteromonas        fsmvvlsslfagwles------kigwqaln-vwsipvvllaf--iasvwfrrrspllpia   388
                                         367
capsulatus               FGGVFLASLSSGGLMTCASADAVAGWQAVN-LAMLPFLTLAGAALIWLVLRPKDTR----   405
Thioalkalivibrio         ftlvaagsllagallr------plgwdgln-lamlpviavltvailwlwrddarappra-   399
Methylobacterium         fgtmavgsfssgglla------hygwdvvl-wvsfgplavavaalalaaasr-papaag-   406
Bordetella               fglmalgsfssgglls------aygwntvl-wvsfvplvvaavalvlvpsrl-lsgrage   402
Ochrobactrum             FGTVACASFFAGSLLH------SSGWETIN-WLVFPIVALVLVPLILR---LKPKGAAAE   397
Pseudomonas              fgtvavasfgsgrlln------tsgwetin-glmlplialvlallgwlawrnrrqsaaaa   400
                         *  :   :  :* :          .*  :


Thalassospira            ---   395
Synechococcus            ---   397
Halomonas                g--   392
Vibrio                   ---   389
Pseudoalteromonas        dkp   391
capsulatus               ---   405
Thioalkalivibrio         ---   399
Methylobacterium         ---   406
Bordetella               gek   405
Ochrobactrum             A--   398
Pseudomonas              ap-   402
```

Figure S2. Alignments of CcoA amino acid sequences among Proteobacterial species. The CcoA sequences are from *Rhodobacter capsulatus* (**capsulatus**) from Rhodobacterales, *Methylobacterium platani* (Methylobacterium) and *Ochrobactrum anthropi* (ochrabactrum) from Rhizobiales, *Bordetella pertussis* (Bordetella) from Burkhorderiales, *Pseudomonas aeruginosa* (Pseudomonas) from Pseudomonales, *Thalassospira lucentensis* (Thalassospira) from Rhodospirales, *Vibrio orientalis* (Vibrio) from vibrionales, *Halomonas desiderata* (Halomonas) from Oceanospiralles, *Synechococcus* sp. (Synechococcus) from Synecchocales, *Pseudoalteromonas luteoviolacea* (Pseudoalteromonas) from Alteromonadales, *Thioalkalivibrio versutus* (Thioalkalivibrio) from Chromatiales. The Met, His and Cys residues of interest are highlighted in yellow, green, and purple, respectively.
