# Supplementary material for: Cysteine Mutants of the Major Facilitator Superfamily-Type Transporter CcoA Provide Insight into Copper Import
Source: mBio. 2021 Jul 20;12(4):e01567-21. doi: 10.1128/mBio.01567-21 (PMC8406296; doi:10.1128/mBio.01567-21)
Supplement: TABLE S3 [file mbio.01567-21-st003.docx]

| Residues | Outward-open  CcoA_YajR_ | Occluded  CcoA_LacY_ | Inward-open  CcoA_GlpT_ |
| --- | --- | --- | --- |
| C_109_-C_247_ | 39 | 36 | 27 |
| C_109_-C_49_ | 12 | 14 | 14 |
| C_109_-C_367_ | 32 | 28 | 22 |
| C_49_-C_367_ | **22** | 16 | ^a^**10** |
| C_49_-C_247_ | **32** | 23 | ^a^**16** |
| C_367_-C_247_ | 21 | 22 | 19 |

**Table S3.** Approximate distances (Å) separating the Cys residues αC—αC of CcoA in its homology models with different conformations.

^a^ Shortening distances between C_49_~C_247_ and C_49_~C_367_ during outward-open to inward-open conformational transition are indicated in bold.
