# Supplementary material for: Cysteine Mutants of the Major Facilitator Superfamily-Type Transporter CcoA Provide Insight into Copper Import
Source: mBio. 2021 Jul 20;12(4):e01567-21. doi: 10.1128/mBio.01567-21 (PMC8406296; doi:10.1128/mBio.01567-21)
Supplement: TABLE S2 [file mbio.01567-21-st002.docx]

**Table S2: Primers used in this study**

| Primers | Sequence from 5’ to 3’ |
| --- | --- |
| M265A-F  M265A-R | ([3](#_ENREF_3)) |
| M30A-F  M30A-R | GTTTCTGGGCGCGCAGGCGTCGATGATCTTCACC  GGTGAAGATCATCGACGCCTGCGCGCCCAGAAAC |
| M32A-F  M32A-R | GGCGCGCAGATGTCGGCGATCTTCACCGTCGG  CCGACGGTGAAGATCGCCGACATCTGCGCGCC |
| M69A-F  M69A-R | TGACGGCGCAGCCGGCGTCGTCCTTCATGG  CCATGAAGGACGACGCCGGCTGCGCCGTCA |
| M73A-F  M73A-R | CCGATGTCGTCCTTCGCGGCGGTTTACGGCCG  CGGCCGTAAACCGCCGCGAAGGACGACATCGG |
| M227A-F  M227A-R | GGCGATGATCTGCGGCGCGGTTTCCTATGCGCTG  CAGCGCATAGGAAACCGCGCCGCAGATCATCGCC |
| H249A-F  H249A-R | GGTGGGCTGCGGCGCCACCACGACGAAT  ATTCGTCGTGGTGGCGCCGCAGCCCACC |
| H274A-F  H274A-R | CCTCTTTCTTCACCGGCGCTCTGATTGCCCGCTTTG  CAAAGCGGGCAATCAGAGCGCCGGTGAAGAAAGAGG |
| C49A-F  C49A-R | GGCGACGAACCCCGCTCTGGCGACGCTG  CAGCGTCGCCAGAGCGGGGTTCGTCGCC |
| C109A-F  C109A-R | CTTTCCCGCTGTTCGCCCTCGGCTCGCTTC  GAAGCGAGCCGAGGGCGAACAGCGGGAAAG |
| C225A-F  C225A-R | CCGGGTGGCGATGATCGCCGGCATGGTTTCCTAT  ATAGGAAACCATGCCGGCGATCATCGCCACCAGG |
| C247A-F  C247A-R | GCGGTGGTGGGCGCCGGCCACACCAC  GTGGTGTGGCCGGCGCCCACCACCGC |
| C367A-F  C367A-R | CGGGCTGATGACCGCCGCCTCGGCCGAT  ATCGGCCGAGGCGGCGGTCATCAGCCCG |
